# Pleomorphic adenomia of lower lip: A case report

**DOI:** 10.12669/pjms.39.6.7191

**Published:** 2023

**Authors:** Muhammad Usman Akram, Nausheen Hasham, Hafsa Atique, Ahmad Raza

**Affiliations:** 1 Muhammad Usman Akram, FCPS, MRCSed, MRCS Assistant Professor Akbar Niazi Teaching Hospital and (IMDC), Islamabad, Pakistan; 2 Nausheen Hasham, MBBS Akbar Niazi Teaching Hospital and (IMDC), Islamabad, Pakistan; 3 Hafsa Atique, MBBS, Medical Student Akbar Niazi Teaching Hospital and (IMDC), Islamabad, Pakistan; 4 Ahmad Raza, FCPS, MRCS Professor Akbar Niazi Teaching Hospital and (IMDC), Islamabad, Pakistan

**Keywords:** Pleomorphic adenoma, Minor salivary glands, Lower lip tumors, Lip pleomorphic adenoma

## Abstract

Pleomorphic adenoma is a benign tumor of the salivary glands. It commonly occurs in the parotid gland, palate, upper lip and cheek. The authors present a rare case of a pleomorphic adenoma of the lower lip in a 30 years old female admitted on 20^th^ of July, 2022 at Akbar Niazi Teaching Hospital, Islamabad with a complaint of painless, slightly itchy swelling on the lower lip for the last four months. Careful history and examination revealed a swelling of the lower lip which had gradually increased in size but was static for the last three months. As the patient complained of cosmetic and social inconvenience, it was surgically managed. Any post-operative complications were ruled out and the patient was sent home in a good condition. Much research is warranted to know the exact etiopathogenesis and appropriate management of pleomorphic adenoma of the lower lip.

## INTRODUCTION

The salivary glands are important components of the faciomaxillary area and are divided into major and minor glands. Tumors of the salivary glands make up 3-10% of all the head and neck tumors. Nearly 80% of these tumors are benign and involve the major salivary glands, whereas malignant tumors are seen typically in minor salivary glands. Thus, the potential of malignancy is inversely proportional to the size of the salivary glands.^1^

Pleomorphic adenoma (PA) is one of the most common benign tumors of the salivary glands. It usually affects the major salivary glands, having an incidence of 84% in the parotid glands, 8% in the submandibular glands and 1% in the sublingual glands. Scarcely does it involve the minor salivary glands with an incidence of 6.4%.^2^ Also referred to as benign mixed tumor, pleomorphic adenoma namely suggests a varied histological figure. It consists of a diversity of differentiated cells, ranging from epithelial descent of ductal and non-ductal cells and other cells including hyaline, cartilaginous, myxoid and osseus type of mesenchyme-like cells. This contrast in cell types leads to difficult diagnosis of the lesion.^3^

History of smoking, intake of cholesterol-rich diet and exposure of the head and neck regions to radiation are signification risk factors for pleomorphic adenoma.^4^ This type of adenoma is generally seen in the third to fifth decade of life; predominantly females. Parotid gland is the most common extra-oral site for this tumor. Among the intraoral sites involved, the palate is the most common (42.8-68.8%), then the upper lip (10.1%) and cheek (5.5%). Other sites with limited involvement include the oropharyngeal isthmus (2.5%), retromolar trigone (0.7%), floor of mouth and the oral mucosa.^5^

Labial pleomorphic adenomas account for 20-40% of the total intra-oral incidences. They usually involve the upper lip and rarely do they occur in the lower lip, i.e., ≤ 3% amongst all intraoral pleomorphic adenomas.^6^ Patients with pleomorphic adenoma commonly present with an asymptomatic, painless, slow-growing firm nodular mass, which tends to be single and mobile. This mass mostly has an intact epithelial covering and is scarcely complicated with mucosal ulceration and paresthesia. If neglected, the mass can gradually increase in size and involve the overlying skin or mucosa. Grossly, it appears as a well-circumscribed, round to oval mass, which is smooth in consistency.

The differential diagnosis of a localized mass in the lower lip includes mucocele, traumatic fibromas, or focal fibrous hyperplasia, pleomorphic adenoma, benign epithelial salivary gland tumor and basal cell adenoma.^6^ Hereunder, we present a case report of a rare presentation of pleomorphic adenoma of the lower lip, near the midline. Informed patient consent was obtained for the drafting of this report.

## CASE PRESENTATION

A 30 years old female presented to surgical outpatient department (OPD) of Akbar Niazi Teaching Hospital (ANTH), Islamabad on 20^th^ of July, 2022 with a complaint of painless, slightly itchy swelling on the lower lip for the last four months. Careful history and examination revealed a swelling of the lower lip which had gradually increased in size but was static for the last three months. There was no associated history of fever, pain, bleeding or difficulty in chewing. History of diabetes, hypertension, smoking or tobacco chewing was not present. The patient was not on any past medications or treatment. Her family history was also insignificant.

**Fig.1:**
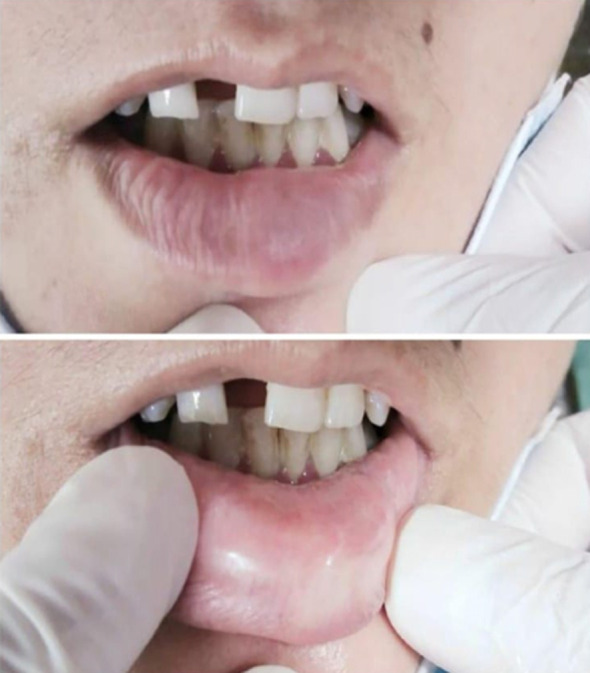
Preoperative swelling at lower lip.

On local examination, the swelling was 1x1 cm, towards the left of the midline (Fig.1). Its surface was smooth and firm and was mobile and non-tender. The adjacent lymph nodes were not palpable. The ear, nose and throat examination did not reveal any signs of tonsillar enlargement, epistaxis or presence of any visible mass. Systemic examination also did not reveal anything of clinical significance. The patient had presented on her primary visit to the doctor due to cosmetic disfigurement of the face and social apprehension. Thus, she was admitted and surgery was planned.

Investigations included all the baseline tests and COVID-19 profile. CT scan face/ maxillofacial and neck showed a well-defined, benign appearing, soft-tissue subcutaneous mass on the lower lip, left to the midline. Fine needle aspiration cytology (FNAC) revealed presence of ductal epithelial cell clusters in the background of a chondromyxoid matrix with scattered myoepithelial cells. Anesthesia fitness test was done and the patient was prepared for surgery. Consent was taken and possible risks and complications were explained to the patient.

**Fig.2:**
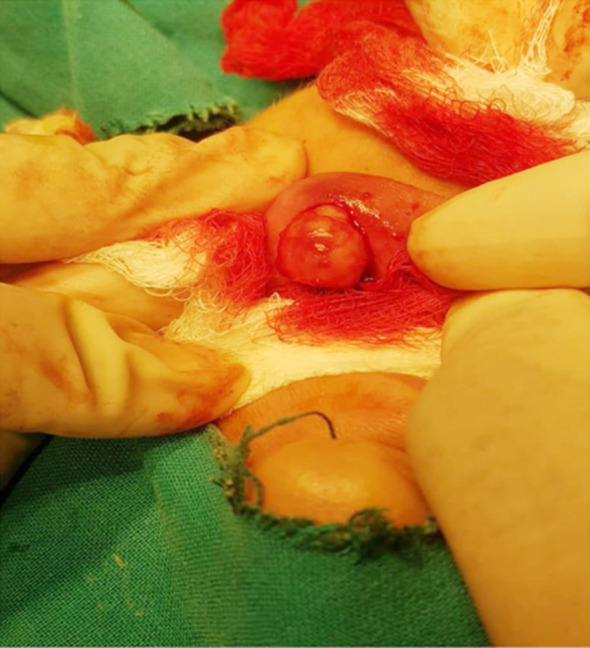
Intraoperative exposure of swelling.

**Fig.3:**
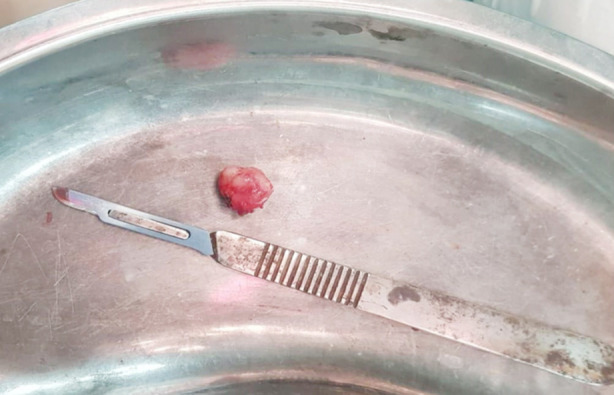
Excised swelling.

**Fig. 4:**
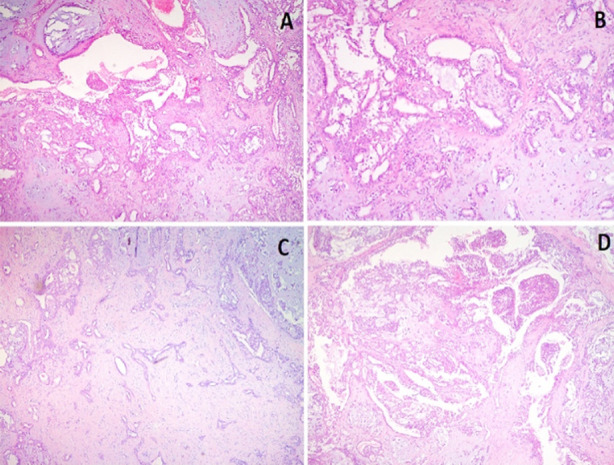
A & B, epithelial component glandular structures and C & D, squamous metaplastic changes.

The surgical procedure was conducted under general anesthesia. The surgical site was identified and elliptical incision around the swelling was made. The skin and underlying tissue were separated (Fig.2). The mass was then excised with a wide local excision and sent for histopathology (Fig.3). The incised wound was closed with absorbable suture (Vicryl 4.0). According to histopathology report pleomorphic adenoma was diagnosed having features of epithelial component glandular structures and squamous metaplastic changes were seen in specimen and no evidence of malignancy (Fig. 4). The patient was discharged on 1st postoperative day and after one week patient was called in surgical OPD for follow-up, the surgical site was examined and no anomalies were found so she had been followed after three months. Clinically finding of no recurrence were found.

## DISCUSSION

Worldwide, cancer of the oral cavity is one of the top ten most malignant neoplasms. Southeast Asia is reported to have the highest incidence of these cancers, mainly owing to the habitual habit of the locals of smoking, alcohol consumption and chewing betel quid.^7^ A study was conducted by Waqar et al, there was a male predominance, with tumors of the oral cavity being the most common and 3% of them occurring in the salivary gland.^8^ The West is reported to have a prevalence of 2.5-3 per 100,00 people per year of salivary gland tumors. They have a lower incidence of malignancy, with approximately 80% of them being benign.^6^

Study by Zaman et al found a ratio of 2.6:1:1.3:0.05 of the tumors in the parotid (51.6%), submandibular (19.7%), minor salivary glands (27.4%), and the sublingual gland (1%); in contrast to the traditional ratio of 1:0.1:0.1:0.01, thus showing a relatively higher incidence in the submandibular and minor salivary glands. It also concluded that pleomorphic adenoma; a benign lesion, was the most commonly diagnosed tumor (47.2%) in the study population, followed by adenoid cystic carcinoma (17.5%), mucoepidermoid carcinoma (16.4%) and basal cell adenoma (4.3%).^9^

A study conducted in Jammu Kashmir reinforces this statement. Histopathological evidence concluded that 51% of the samples collected were benign and amongst them, pleomorphic adenoma was the most frequent diagnosis on biopsy.^10^ Although pleomorphic adenoma is a benign and well-defined tumor, its morphological interpretation can be difficult. FNA cytology is usually used as a first line diagnostic test for any salivary gland lesion. However, the myoepithelial cells are difficult to diagnose accurately even on cytology, given their mimicking behavior and the heterogenous appearance of the tumor.^11^ Thus, histopathology is warranted.

The afore mentioned report is about a pleomorphic adenoma of the lower lip. A similar rare case was recorded in literature by Nourwali et al.^6^ In this study, 26 years old male presented with swelling lower lip and diagnosis were made clinically and excision biopsy was performed. Features of pleomorphic adenoma was confirmed in histopathology. Another case was reported by Ahmedi et al in which 10 years old girl was presented with similar swelling at upper lip which was also confirmed as pleomorphic adenoma on histopathology after excision biopsy.^12^ As stated by a study in Nigeria, the treatment of choice for pleomorphic adenoma is surgical excision.^2^ Our management plan coincides with this study. Although recurrence of pleomorphic adenoma is rare, with a rate of 2-44%, the failure of proper excision of the lesion can play a critical role in it.^13^ Thus, a proper surgical technique is needed to avoid any such complications.

## CONCLUSION

Pleomorphic adenoma is a benign and common lesion amongst all the salivary gland tumors. However, its occurrence on the lower lip is quite rare and only a few cases have been reported over the years. Thus, much research is warranted to know its exact etiopathogenesis and appropriate management.

### Authors’ Contribution:

**MUA:** provided concept/design, acquisition of data.

**NN & AR:** did project management.

**HA & AR:** did editing of manuscript and project management.

**MUA:** takes the responsibility and are accountable for all aspects of the work in ensuring that questions related to the accuracy or integrity of any part of the work are appropriately investigated and resolved.
